# Well-being in trainee and faculty physicians

**DOI:** 10.1080/10872981.2021.1950107

**Published:** 2021-07-12

**Authors:** Gary N. Frishman, Christina A. Raker, David Frankfurter

**Affiliations:** a Professor of Obstetrics and Gynecology, Division of Reproductive Endocrinlogy and Infertility, Department of Obstetrics and Gynecology, Warren Alpert Medical School of Brown University, Providence, RI, USA; b Division of Research, Department of Obstetrics and Gynecology, Warren Alpert Medical School of Brown University, Providence, RI, USA; c Professor of Obstetrics and Gynecology, Division of Reproductive Endocrinlogy and Infertility, Department of Obstetrics and Gynecology. George Washington University School of Medicine and Health Sciences, Washington, DC, USA

**Keywords:** Physician wellness, burnout, duty hours, workplace environment

## Abstract

**Background:** Physician well-being remains a critical topic with limited information concerning the impact of the progression of training and duty hours. To date, our knowledge and interventions have not adequately addressed these issues. We assessed differences in well-being across the USA: (1) between all post-graduate trainees and their academic core faculty; (2) between all obstetrics and gynecology trainees and their academic core faculty and (3) during the progression of training within obstetrics and gynecology (OB/GYN).**Methods:** A cross-sectional study analyzing responses to well-being questions included in the 2017–2018 Accreditation Council for Graduate Medical Education (ACGME) surveys given to all U.S. trainees and core faculty. **Results:** More than 85% of all U.S. physician-trainees and faculty surveyed responded. Respondents included 128,443 trainees from all specialties combined, 5,206 OB/GYN residents and 799 OB/GYN subspecialty fellows. A total of 94,557 faculty from all specialties combined, 4,082 general OB/GYN faculty and 1,432 sub-specialty OB/GYN faculty responded. Trainees were more negative than faculty for the majority of questions for both all trainees combined and within OB/GYN when progressing from resident to subspecialty fellow to subspecialty faculty (p ≤ 0.05). Questions focusing on work satisfaction (e.g., pride in work) were more negative for residents compared to fellows and for fellows compared to faculty. In contrast to work satisfaction, responses to the question ‘Felt the amount of work you were expected to complete in a day was reasonable’ showed either no difference or higher scores for trainees compared to their faculty. **Conclusions:** Although an issue for all physicians, well-being impacts trainees more, and differently, than faculty and well-being improves during training from resident to fellow to faculty. Survey responses suggest that interventions should focus on workplace satisfaction over workplace environment areas and further limitations in duty hours are unlikely to improve physician well-being.

## Introduction

The challenge of physician well-being and burnout is profound. Although definitions vary, burnout has been referred to as the antithesis of wellness. [1] In the USA, the prevalence of burnout among medical students and residents is between 40% and 76%. [2]

Physician burnout negatively influences work satisfaction and personal relationships while increasing the risk of substance abuse and depression. Indeed, 8–12% of practicing physicians may develop a substance abuse problem during their career. [3] A majority of studies report a negative relationship between burnout and multiple measures of productivity. [4] Moreover, physician burnout negatively impacts safety-related quality of care [5] and is associated with higher in-hospital mortality rates. [3] It is estimated that costs related to physician burnout are approximately 4.6 USD billion per year in the USA alone.

In December 2016, the alarming rates of depression and suicide among U.S. health-care workers led the National Academy of Medicine to launch an Action Collaborative on Clinician Well-Being and Resilience[6]. Within Obstetrics and Gynecology (OB/GYN), the Council on Resident Education in Obstetrics and Gynecology (CREOG) initiated the Physician Satisfaction and Wellness Initiative[7]. Despite these interventions, the National Academy of Medicine (NAM), the Association of American Medical Colleges (AAMC), and the Accreditation Council for Graduate Medical Education (ACGME) stated that ‘the problem of clinician burnout, depression and other mental disorders, and suicide [has] reached a crisis level’[8].

The ACGME recognizes the issue of well-being among physicians and the need to improve patient safety, reduce fatigue and decrease burnout [9,10]. In 2017, the ACMGE revised their educational program requirements. A core focus of the new requirements ‘is the philosophy that residency education must occur in a learning and working environment that fosters excellence in the safety and quality of care delivered to patients’ and ‘directly address how Sponsoring Institutions and programs can enhance resident and fellow well-being’[11]. In addition, the ACGME required increased trainee supervision and further revised duty hours regulations. Annually, the ACGME surveys trainees and academic faculty of all ACGME-approved residency and fellowship programs along multiple dimensions. The academic year 2017–2018 marked the first time that the ACGME surveys included questions related to well-being.

The impact of emotional exhaustion, depersonalization and lack of control and social support within trainees on burnout and wellness are well recognized [12,13]. However, efforts to date have not focused on the impact of the distinct environments of workplace environment compared to workplace satisfaction as well as the role of progression of training as it relates to wellness. To help better understand the differences in well-being and assess optimal areas for interventions, we utilized derivative data from the ACGME surveys to assess for differences 1) between all post-graduate trainees and all their academic core faculty; 2) between all obstetrics and gynecology trainees and all their academic core faculty and 3) during the progression of training within obstetrics and gynecology.

## Materials and methods

This cross-sectional cohort study evaluated summarized responses to the well-being questions included in the 2017–2018 ACGME trainee and faculty surveys in this first academic year that these questions were introduced. In these surveys, all faculty which were designated by training programs as ‘core’ were invited to participate and represent the only faculty surveyed by the ACGME. Core faculty have a significant role in trainee education and devote at least 15 hours per week to trainee education and administration. The 2017–2018 survey reports are available to programs via the ACGME website (https://acgme.org/) in the Reports tab and Survey subsection. The ACGME stipulated that the subsequent reports were not to be disseminated or used for research. As such, our analysis utilized the 2017–2018 surveys as the only available data set.

We used aggregated US survey data to assess all trainees (Appendix 1) and faculty (10,153 programs), OB/GYN residents and faculty (269 programs), and fellows and faculty in the four ACGME accredited OB/GYN subspecialties (see below) to evaluate differences in responses to well-being questions between physicians in training and core faculty in practice.

OB/GYN subspecialties include Female Pelvic Medicine and Reconstructive Surgery (50 programs), Gynecologic Oncology (50 programs), Maternal-Fetal Medicine (82 programs), and Reproductive Endocrinology and Infertility (47 programs). To assess changes in well-being during career progression, within OB/GYN, OB/GYN residents’ responses were compared to OB/GYN subspecialty fellows’ responses which were then compared to their OB/GYN subspecialty faculty responses.

For each survey, the overall percentage of surveyed individuals responding was supplied by the ACGME but not broken down by specific question. Each of the 12 well-being survey questions was answered via a five-point Likert scale with options of Never (1), Rarely (2), Sometimes (3), Often (4), and Very often (5). The final two survey questions also included an ‘N/A’ option. To facilitate analysis, the response distribution reported as percentages for each question was converted to frequencies by multiplying each percentage by the total number of respondents. Responses were then binarized as Very Often/Often vs. Sometimes/Rarely/Never to emphasize affirmative responses across questions and between comparison groups. ‘N/A’ responses were excluded from analysis. The survey reports for trainees and faculty for the all training programs as well as those for all OB/GYN programs were each analyzed individually but, given their small size, those from the four OB/GYN subspecialties for fellows and faculty were combined into single sub-specialty groups. The ACGME used the term ‘resident’ to describe both residents and fellows. We used the term ‘trainee’ when these groups were combined and the terms ‘resident’ or “fellow’ where the level of training was clearly ascertainable (e.g., respondents in fellowship training programs).

We used Chi-square or Fisher’s exact test for all comparisons and estimated the absolute difference in proportions as a measure of effect size. Trends in response proportions by career progression were examined by the Cochran–Armitage test for a linear trend versus the null hypothesis of no association. Two-tailed p-values were presented; p < 0.05 was considered statistically significant. Because of the anonymous and public nature of the data used, the Institutional Review Board of Women and Infants Hospital of Rhode Island determined that this study did not meet the criteria for human subjects research and IRB approval was waived.

## Results

In the ACGME 2017–2018 surveys, for all training programs combined a total of 128,443 out of 135,198 (95%) trainees and 94,557 out of 106,922 (88%) faculty responded. Within OB/GYN 95% of residents and 86% of faculty responded to the survey. Similar proportions of OB/GYN subspecialty fellows and faculty responded as well (Table 1). Table 2 displays survey responses according to program type. For all programs combined, when comparing All Trainees to All Faculty regarding ‘Very Often/Often’ responses, trainees scored lower for all questions except for the amount of work expected to be completed in a day. A slightly, albeit significantly, higher percentage of All Trainees felt that the amount of work expected to be completed in a day was reasonable compared to faculty (77.1% vs 74.0% p < 0.001). When comparing OB/GYN residents to OB/GYN faculty, residents similarly scored lower on the majority of the well-being related questions (Table 2). However, there were no differences between OB/GYN residents and faculty regarding whether they felt supported by coworkers, felt the amount of work expected to be completed in a day was reasonable, participated in decisions affecting their work, and knew who to call if something tragic happened. When comparing OB/GYN subspecialty fellows with OB/GYN subspecialty faculty responses, fellows had a lower percentage of Very Often/Often responses to 5 of the 12 questions and a higher percentage of Very Often/Often responses to 4 of the 12 questions. There was no statistical difference between these groups regarding 3 of the 12 questions (Table 2). The only question within the subspecialty groups in which there was >10% absolute difference between these groups was in relation to the feeling of whether the amount of work one was expected to complete in a day was reasonable. Eighty-seven percent of OB/GYN subspecialty fellows responded Very Often/Often to this question compared to 72% of OB/GYN subspecialty faculty (p < 0.001).

**Table 1.** Number of surveyed physicians and percent responded

	Total Surveyed	Total Responded	% Response
All Nationwide Trainees combined	135,198	128,443	95
All Nationwide Faculty combined	106,922	94,557	88
All Obstetrics and Gynecology Residents	5,459	5,206	95
All Obstetrics and Gynecology Faculty	4,773	4,082	86
All Obstetrics and Gynecology Subspecialty fellows	807	799	99
All Obstetrics and Gynecology Subspecialty Faculty	1,591	1,432	90

**Table 2.** Survey responses by program and role

	All Training Programs			Obstetrics and Gynecology Training Programs			Obstetrics and Gynecology Subspecialty Training Programs		
	Trainees	Faculty	P*	Residents	Faculty	P*	Fellows	Faculty	P*
Total number of respondents	128,443	94,557		5,206	4,082		799	1,432	
Please rate how often you have done or experienced each of the following items in the past 3 weeks:	% Very Often/Often			% Very Often/Often			% Very Often/Often		
Reflected on how your work helps make the world a better place	56.6	72.7†	<0.001	54.3	72.5†	<0.001	69.7	75.2	0.005
Felt the vitality to do your work	72.8	86.6†	<0.001	72.1	85†	<0.001	83.7	88.3	0.002
Felt supported by your co-workers	87.2	88.6	<0.001	87.2	87.4	0.79	90.6	89.8	0.54
Was proud of the work you did	81	93.9†	<0.001	83.2	94.2†	<0.001	90.1	95.4	<0.001
Was eager to come back to work the next day	63.5	82.5†	<0.001	60.6	79.8†	<0.001	80.9	84.7	0.019
You felt your basic needs are met	80.6	86.2	<0.001	75.6	83.8	<0.001	91.2	87.4	0.006
You ate well	73.4	83.8†	<0.001	64.3	83.1†	<0.001	87	83.1	0.015
You felt connected to your work in a deep sense	66.7	85.1†	<0.001	67.1	83.6†	<0.001	81	87.3	<0.001
Felt the amount of work you were expected to complete in a day was reasonable	77.1	74	<0.001	70.6	71.7	0.26	87.1	72.3‡	<0.001
Participated in decisions that affected your work	78.1	80.7	<0.001	77.9	77.5	0.63	84.7	81.4	0.048
Had an enjoyable interaction with a patient	89.4	95.7	<0.0001	94.2	97.1	<0.001	96.7	97.8	0.11
Knew who to call when something tragic happened at work	88.8	89.9	<0.001	89.6	90.6	0.16	91.4	91.5	0.95

* Chi-square or Fisher’s exact test.

†Faculty response > trainees’ response by more than 10%

‡ Faculty response < trainees’ response by more than 10%

The majority of respondents in all groups demonstrated a high percentage (>70%) of Very Often/Often response to survey questions (Table 2). The exceptions were how all residents felt their work made the world a better place, their eagerness to come back to work the following day, and whether they felt connected to their work in a deep sense (Table 2). From a faculty perspective, greater than 70% of All Faculty, OB/GYN faculty and OB/GYN subspecialty faculty felt that their work helps make the world a better place. OB/GYN residents mirrored the larger population of All Trainees with 56.6% of All Trainees and 54.3% of OB/GYN residents stating that their work helped make the world a better place. Almost 70% of OB/GYN subspecialty fellows felt their work made the world a better place. While the difference between OB/GYN subspecialty fellows and faculty in response to this question remained significant, it was less pronounced 69.7% vs 75.2%, respectively (p = 0.005). A similar pattern was noted between All Trainees and All Faculty, OB/GYN residents and faculty, and OB/GYN subspecialty fellows and faculty regarding both how eager respondents were to return to work the next day and whether they felt connected to their work in a deep sense (Table 2).

The progression in improvement in well-being from trainee to faculty persisted within Obstetrics and Gynecology when comparing residents to subspecialty fellows to subspecialty faculty (Table 3). However, when analyzing these three groups, two distinct patterns within the workplace emerged. For questions relating to work satisfaction (e.g., vitality to do work or pride in work), there were clear incremental improvements as careers progressed (Resident ≪ Fellow ≪ Faculty). On the other hand, for questions relating to workplace environment (e.g., basic needs met, ate well etc.), although the linear trend was overall still significant with residents scoring the lowest, subspecialty fellows scored higher than their subspecialty faculty in these areas (Resident ≪ Fellow > Faculty).

**Table 3.** Survey responses by progression within obstetrics and gynecology from resident to subspecialty fellow to subspecialty faculty differentiated by workplace satisfaction versus workplace environment

	Residents		Fellows	Sub-specialty Faculty	Residents> fellows >Sub-specialty Faculty
Total number of respondents	5,206		799	1,432	P linear trend*
Please rate how often you have done or experienced each of the following items in the past 3 weeks:	% Very Often/Often				
Work satisfaction					
Reflected on how your work helps make the world a better place	54.3	69.7		75.2	<0.001
Felt the vitality to do your work	72.1	83.7		88.3	<0.001
Was proud of the work you did	83.2	90.1		95.4	<0.001
Was eager to come back to work the next day	60.6	80.9		84.7	<0.001
You felt connected to your work in a deep sense	67.1	81		87.3	<0.001
Workplace environment					
Felt supported by your co-workers	87.2	90.6		89.8	0.002
You felt your basic needs are met	75.6	91.2		87.4	<0.001
You ate well	64.3	87		83.1	<0.001
Felt the amount of work you were expected to complete in a day was reasonable	70.6	87.1		72.3	<0.001
Participated in decisions that affected your work	77.9	84.7		81.4	<0.001
Had an enjoyable interaction with a patient	94.2	96.7		97.8	<0.001
Knew who to call when something tragic happened at work	89.6	91.4		91.5	0.032

* Cochran–Armitage test for linear trend against the null hypothesis of no association.

## Discussion

In this study, we found that trainee and faculty ACGME survey responses were consistent in demonstrating an impairment of well-being with a greater impact on trainees when compared to faculty. Furthermore, within OB/GYN, an important finding is that when comparing the 3 stages of training from resident to subspecialty fellow to faculty, the more junior roles had statistically more concerning scores for well-being. A critical concept is that responses suggest that well-being is more impacted by workplace satisfaction than the workplace environment. Although other studies have also noted the important concept of ‘meaning’ in wellbeing [14,15] our study is important in that it describes and differentiates the domains of workplace satisfaction and workplace environment.

The only question where all trainees, regardless of level of training, had the same or more ‘Very Often/Often’ responses compared to their respective faculty was to the question ‘Felt the amount of work you were expected to complete in a day was reasonable’. This suggests that the absolute workload/hours is not the core issue and that work satisfaction influences well-being more than workplace environment.

There are multiple theories as to the etiology of burnout which help explain our findings. Panagioti reported that key causes of burnout include a lack of control over one’s job, prolonged work stress and an imbalance between job demands and skill sets [16]. These conclusions reflect the life experience of a trainee and can help explain the disparity between trainee and faculty. Our findings suggest that well-being is less a basic needs issue but, rather, a higher level concern about how one perceives their role in medicine. This ‘beating your head against the wall’ mindset was especially evident in resident responses on how trainees felt that they made the world a better place, how eager they were to return the next day, and whether they felt a deep connection to their work (Table 2).

It is necessary to consider the contributors that impact well-being in order to help direct remedies. Notably, organizations have increased physicians’ regulatory and administrative responsibilities without a proportional increase in organizational support [17]. Such well-being stressors point to the need for culture change and organizational interventions. This approach is echoed by the findings of a recent meta-analysis that concluded that structural organizational strategies, such as locally developed modifications to the clinical work processes, can positively impact physician burnout [18]. Another meta-analysis on interventions to reduce physician burnout also reported that burnout is a systems level issue. These findings emphasize that reducing burnout requires organizational level intervention programs rather than ones targeting individual providers [16]. Our conclusions are consistent with this in that OB/GYN residents, subspecialty fellows and faculty all felt supported by their co-workers yet show signs of impaired well-being.

Our findings, which reflect the inclusion of all training programs across the country, should not diminish the importance of interventions towards well-being at the individual level. However, there needs to be an emphasis on systemic efforts at the organizational level. Suggestions for improving the organizational workflow and addressing institutional-based factors that contribute to burnout include adding advanced practice providers to help with the work flow (allowing providers to practice at the highest level of their license), improving and optimizing electronic medical record systems (specifically order entry and documentation support) and the use of medical scribes [19].

Limitations of this study include the heterogeneous nature of the survey recipients within their fields. Specifically, the survey is applied to all types of training programs and, within specialties, faculty varied (e.g., Internal Medicine includes Invasive Cardiologists, Primary Care Internists, Nephrologists, etc.). Likewise, OB/GYN faculty include Specialists in Obstetrics and Gynecology, providers practicing Obstetrics only, Gynecology only, etc. and the subspecialty groups combined the 4 ACGME accredited OB/GYN subspecialties. That being said, our focus was the stage and degree of specialization of training and not the specific field of study or practice. Importantly, all studies assessing physician well-being have similarly blended populations. Although a validated survey tool was not used, we believe the consistency within the data and targeted focus of the questions is a strength. Furthermore, these were the questions chosen to assess well-being by the governing body for residency training. In addition, the use of the uniform ACGME surveys allows an ‘apples to apples’ approach when comparing trainees to faculty across the entire nation. To our knowledge, such a comparison has never been done. We could not expand beyond one year due to restrictions placed by the ACGME and the study of subsequent years is not possible. As such, any changes with time and/or the influence of the pandemic with its impact on culture and medicine are not able to be investigated. Reports on burnout during the pandemic note the importance of institutional support but differ on the value of mindfulness techniques [20,21]. We also cannot definitively conclude how responders interpreted questions such as the role of peer support or eating well and combined questions into the categories of workplace satisfaction and workplace environment based on our interpretation of the questions’ intent.

Strengths of this study include the exceptionally high survey response rate, 86–99%, for both trainees and faculty. We believe this is the first study to highlight how the differences in well-being, by stage of training, are impacted by the distinctive domains of work satisfaction compared to workplace environment. This may encourage a targeted approach for each area and can help guide strategy and policy decisions on how to positively influence well-being.

In summary, physician well-being remains a vital topic in medicine. There are clear differences in well-being across levels of experience with trainees being more negatively impacted than faculty. Our findings suggest that further reductions in work hour rules are unlikely to meaningfully improve well-being. Work satisfaction plays a greater role in well-being than workplace environment. It is important to include systems and institutional level interventions when implementing measures to improve well-being.

## Data Availability

The datasets are available for programs from the ACGME and are available from the authors if desired. An example of a data set is provided as Appendix 1.

## Appendix 1.

**2017–2018 ACGME Resident Survey**

USA – Aggregated Program Data Well-Being Survey Questions

**Survey taken: January 2018 – April 2018**

Programs Surveyed 10,153

Residents Responded 128,443 /

135,198

Response Rate 95%

In July 2017, the ACGME implemented the revised Section VI of the Common Program Requirements. At the heart of the new requirements is the philosophy that residency education must occur in a learning and working environment that fosters excellence in the safety and quality of care delivered to patients both today and in the future. An important corollary is that physician well-being is crucial to deliver the safest, best possible care to patients. This year of data collection, which will serve as a baseline measure, aggregate reports will be provided to the program and sponsoring institution. These data will not be provided to the Review Committees to make accreditation decisions.

**Please rate how often you have done or experienced each of the following items in the past 3 weeks**:

**Table:**

	Never (1)	Rarely (2)	Sometimes (3)	Often (4)	Very Often (5)	National Mean
Reflected on how your work helps make the world a better place	3.4%	10.2%	29.8%	31.5%	25.2%	3.6
Felt the vitality to do your work	0.9%	4.7%	21.6%	42.0%	30.8%	4.0
Felt supported by your co-workers	0.5%	1.9%	10.4%	35.7%	51.5%	4.4
Was proud of the work you did	0.3%	2.1%	16.6%	40.0%	41.0%	4.2
Was eager to come back to work the next day	2.5%	8.2%	25.8%	36.7%	26.8%	3.8
You felt your basic needs are met	0.6%	3.1%	15.7%	40.4%	40.3%	4.2
You ate well	0.7%	5.8%	20.1%	36.3%	37.1%	4.0
You felt connected to your work in a deep sense	1.6%	7.0%	24.7%	36.5%	30.3%	3.9
Felt the amount of work you were expected to complete in a day was reasonable	0.7%	3.5%	18.7%	43.1%	34.0%	4.1
Participated in decisions that affected your work	1.2%	4.1%	16.6%	40.0%	38.1%	4.1

**Please rate how often you have done or experienced each of the following items in the past 3 weeks**:

**Table:**

	Never (1)	Rarely (2)	Sometimes (3)	Often (4)	Very Often (5)	N/A	National Mean
Had an enjoyable interaction with a patient	0.2%	1.0%	9.1%	27.6%	59.3%	2.7%	4.5
Knew who to call when something tragic happened at work	1.0%	1.9%	6.5%	22.0%	52.7%	15.9%	4.5

© 2018 Accreditation Council for Graduate Medical Education (ACGME). Percentages may not add to 100% due to rounding.
